# PDI-mediated *S*-nitrosylation of DRP1 facilitates DRP1-S616 phosphorylation and mitochondrial fission in CA1 neurons

**DOI:** 10.1038/s41419-018-0910-5

**Published:** 2018-08-29

**Authors:** Duk-shin Lee, Ji-Eun Kim

**Affiliations:** Department of Anatomy and Neurobiology, Institute of Epilepsy Research, College of Medicine, Hallym University, Chuncheon, 24252 South Korea

## Abstract

Dynamin-related protein 1 (DRP1) is a key molecule to regulate mitochondrial fission. DRP1 activity is modulated by phosphorylation and *S*-nitrosylation on serine and cysteine residues, respectively. However, it is still unexplored whether *S*-nitrosylation of DRP1 affects its phosphorylation. In the present study, we found that *N*^ω^-nitro-l-arginine methyl ester hydrochloride (l-NAME, a NOS inhibitor) abolished *S*-nitrosylated (SNO-DRP1) and DRP1-serine (S) 616 phosphorylation levels in CA1 neurons under physiological condition. l-NAME led to mitochondrial elongation. In spite of the sustained NO synthesis, status epilepticus (a prolonged seizure activity, SE) diminished SNO-DRP1 and DRP1-S616 levels in CA1 neurons, accompanied by the reduced protein disulfide isomerase (PDI) expression and mitochondrial elongation. SE did not influence thioredoxin 1 (Trx1, a denitrosylating enzyme) activity, which was unaffected by l-NAME under physiological and post-SE condition. PDI knockdown decreased SNO-DRP1 and DRP1-S616 levels concomitant with mitochondrial elongation in CA1 neurons without altered NO synthesis under physiological condition. These findings indicate that PDI may be a NO donor of DRP1 to regulate DRP1-S616 phosphorylation, independent of Trx1 activity. Therefore, we suggest that PDI-mediated *S*-nitrosylation of DRP1 may be one of the major regulatory modifications for mitochondrial dynamics.

## Introduction

Mitochondria are key intracellular energy-generating organelles with various cellular functions and a major source of cellular reactive oxygen species (ROS)^1^. Since mitochondria are highly dynamic organelles that frequently fuse and divide (referred to as fusion and fission, respectively)^2^, the machineries of these dynamics are responsible for the shape, size, number, distribution, and function of mitochondria^3^. Thus, balanced mitochondrial dynamics are important for sustaining mitochondrial functions^4^. Mitochondrial fusion is regulated by mitofusin-1 and -2 (MFN1 and MFN2) and optic atrophy 1 (OPA1), while fission is mediated by dynamin-related protein 1 (DRP1)^5^. DRP1 is recruited to the constricted mitochondrial membrane and fragments mitochondria in a GTPase-dependent manner^6^, which is mainly regulated by phosphorylation^2,7^. Briefly, phosphorylation of DRP1 at serine 616 (S616) site activates DRP1-mediated mitochondrial fission^8,9^, while phosphorylation of serine 637 (S637) inhibits mitochondrial fission by diminishing its attachment on mitochondria^10–12^. Recently, it has been reported that impaired DRP1-mediated mitochondrial fission results in necrotic neuronal death due to dysfunctions of mitochondrial re-localization and ATP synthesis/supply^13–17^. In contrast, intensified DRP1 activation also contributes excessive fragmentation and concomitant apoptosis^18,19^. Thus, the maintenance of proper DRP1 activity is one of the important factors for cell viability.

Nitric oxide (NO) is the first gaseous second messenger and plays an important role in vasodilation^20^. NO also reacts with sulfhydryl (-SH, thiol) groups of proteins and leads to the formation of *S*-nitrosothiols (-SNO), so-called *S*-nitrosylation^21,22^. *S*-nitrosylation regulates enzymatic activity of target proteins via modulation of active site cysteine (C) residues or via allosteric changes of protein structures^23,24^. Interestingly, nitrosative stress causes aberrant *S*-nitrosylation of DRP1 to increase its enzyme activity, which leads to excessive mitochondrial fragmentation^25–27^. Thus, nitrosative stress-mediated mitochondrial fragmentation plays an important role in many neurodegenerative disorders including Parkinson’s disease, Huntington’s disease, amyotrophic lateral sclerosis, and stroke^28^. Unlike these neurodegenerative diseases, we previously reported that the impaired DRP1-mediated mitochondrial fission induces programmed necrotic neuronal death in hippocampal CA1 neurons vulnerable to status epilepticus (SE, a prolonged seizure activity), independent of NO synthase (NOS) activity^13,14,29^. Furthermore, NO triggers DRP1 phosphorylation at S616, which results in its activation and recruitment to mitochondria, independent of DRP1 *S*-nitrosylation^30^. Therefore, whether *S*-nitrosylation of DRP1 affects aberrant mitochondrial dynamics during seizure-induced neuronal necrosis and pathophysiology of epilepsy remains to be answered.

Protein disulfide isomerase (PDI) is one of chaperones in the endoplasmic reticulum (ER), and also present in cytoplasm as well as cell surface, which mediates protein folding through thiol–disulfide exchange^31–33^. Recently, we have reported that PDI reduces seizure threshold via sulfhydration (reduction) of disulfide bonds on N-methyl-d-aspartate receptor (NMDAR)^34^. Since *S*-nitrosylation is a redox-based post-translational modification^35–37^, it is simply expected that PDI would be also aberrantly *S-*nitrosylated under pathophysiological conditions, leading to neuronal death^38–41^. Furthermore, *S*-nitrosylated (SNO-) PDI acts as a transporter for NO residues^42^. Thus, it is noteworthy whether PDI mediates *S*-nitrosylation on DRP1, which could regulate mitochondrial dynamics during neuronal degeneration. Here, we demonstrate that *N*^ω^-nitro-l-arginine methyl ester hydrochloride (l-NAME, a NOS inhibitor) abolished SNO-DRP1 and DRP1-S616 levels in CA1 neurons. l-NAME also elongated mitochondrial length under physiological condition, while it could not affect DRP1 expression. In spite of the prolonged NO synthesis, SE significantly diminished SNO-DRP1 and DRP1-S616 levels in CA1 neurons, accompanied by the reduced PDI expression and the mitochondrial elongation. Similarly, PDI knockdown decreased SNO-DRP1 and DRP1-S616 levels without altered NO synthesis under physiological condition. Furthermore, PDI siR`NA evoked mitochondrial elongation in CA1 neurons. To our knowledge, this is the first study to show that PDI may be a NO donor of DRP1 to regulate DRP1-S616 phosphorylation. Therefore, our findings suggest that PDI-mediated *S*-nitrosylation of DRP1 may be one of major regulatory modifications for mitochondrial fission.

## Results

### l-NAME induces mitochondrial elongation in the CA1 neurons under normal condition

**Fig. 1:**
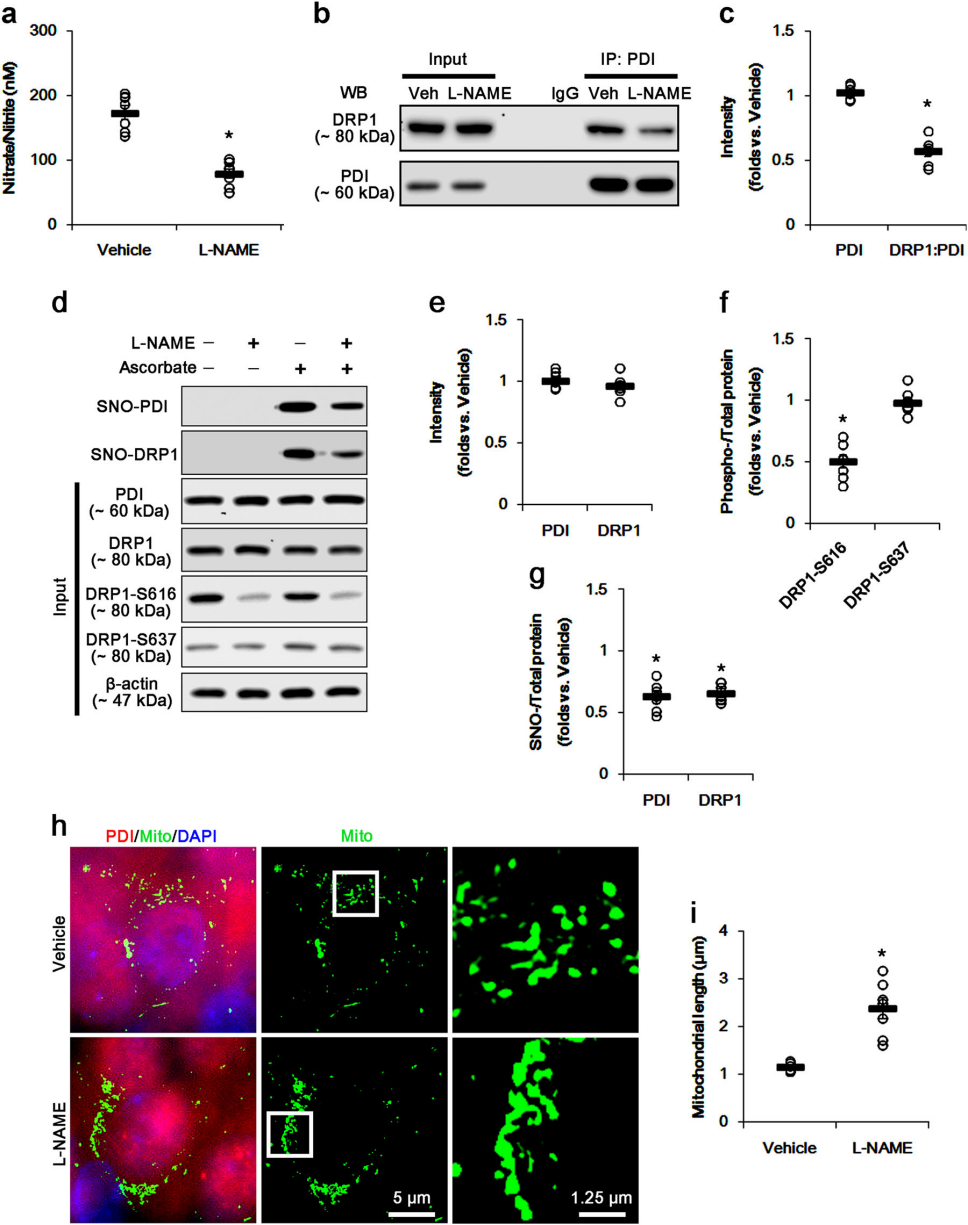
The effects of l-NAME on SNO-PDI, SNO-DRP1, and mitochondrial fusion in CA1 neurons under normal condition. l-NAME inhibits NO generation and the binding of PDI to DRP1, and reduces SNO-PDI and SNO-DRP1 levels without changed PDI and DRP1 expressions. However, l-NAME elongates mitochondrial length in CA1 neurons, accompanied by elevated DRP1-S616 phosphorylation. **a** Quantitative values (mean ± S.E.M) of nitrate/nitrite levels in the hippocampus 7 days after l-NAME infusion. Open circles indicate each individual value. Horizontal bars indicate mean value. Error bars indicate SEM (*p < 0.05 vs. non-SE animals; *n* = 7, respectively). **b** Representative western blot for co-immunoprecipitation of PDI interaction with DRP1 following l-NAME treatment. **c** Quantification of co-immunoprecipitation analyses of PDI interactions with DRP1 following l-NAME treatment. Open circles indicate each individual value. Horizontal bars indicate mean value. Error bars indicate SEM (*p < 0.05 vs. vehicle; *n* = 7, respectively). **d** Representative western blot for expression, *S*-nitrosylation and phosphorylation levels of PDI and DRP1 following l-NAME treatment. **e**–**g** Quantification of values (mean ± S.E.M) of expression, phosphorylation, and *S*-nitrosylation levels of PDI and DRP1 following l-NAME treatment. Open circles indicate each individual value. Horizontal bars indicate mean value. Error bars indicate SEM (*p < 0.05 vs. vehicle; *n* = 7, respectively). **h** Quantification of values (mean ± S.E.M) of mitochondrial length in CA1 neurons following l-NAME treatment. Open circles indicate each individual value. Horizontal bars indicate mean value. Error bars indicate SEM (*p < 0.05 vs. vehicle; *n* = 7, respectively). **i** Representative immunofluorescent photo for PDI and mitochondria (Mito) in CA1 neurons. The right panels are the high magnification photos of the rectangles in the middle panel

To investigate whether *S*-nitrosylation of DRP1 affects DRP1-S616 phosphorylation and mitochondrial dynamics, we applied l-NAME to control animals. As compared to vehicle, l-NAME decreased nitrate/nitrite concentration in the CA1 region (*p* < 0.05, Fig. 1a). Although l-NAME could not change PDI and DRP1 expression levels, it diminished ~50% reduction in the binding of PDI to DRP1 in CA1 neurons (*p* < 0.05 vs. vehicle; Fig. 1b, c and Supplementary figure S1). In addition, l-NAME reduced SNO-PDI, SNO-DRP1, and DRP1-S616 levels (*p* < 0.05 vs. vehicle; Fig. 1d, e and Supplementary figure S1), while it did not affect DRP1-S637 level (Fig. 1d–g and Supplementary figure S1). Since the elevated DRP1-S616 phosphorylation accelerates mitochondrial fission^13,19,43^, we measured the mitochondrial length to evaluate the effect of l-NAME on DRP1 activity. l-NAME effectively elongated mitochondrial length to ~2.5-fold of vehicle level in CA1 neurons (*p* < 0.05 vs. vehicle; Fig. 1h, i). These findings indicate that NO may participate in the mitochondrial fission via *S*-nitrosylation and S616 phosphorylation of DRP1 under physiological condition.

### l-NAME inhibits prolonged NO synthesis without changed seizure activity in response to pilocarpine

**Fig. 2:**
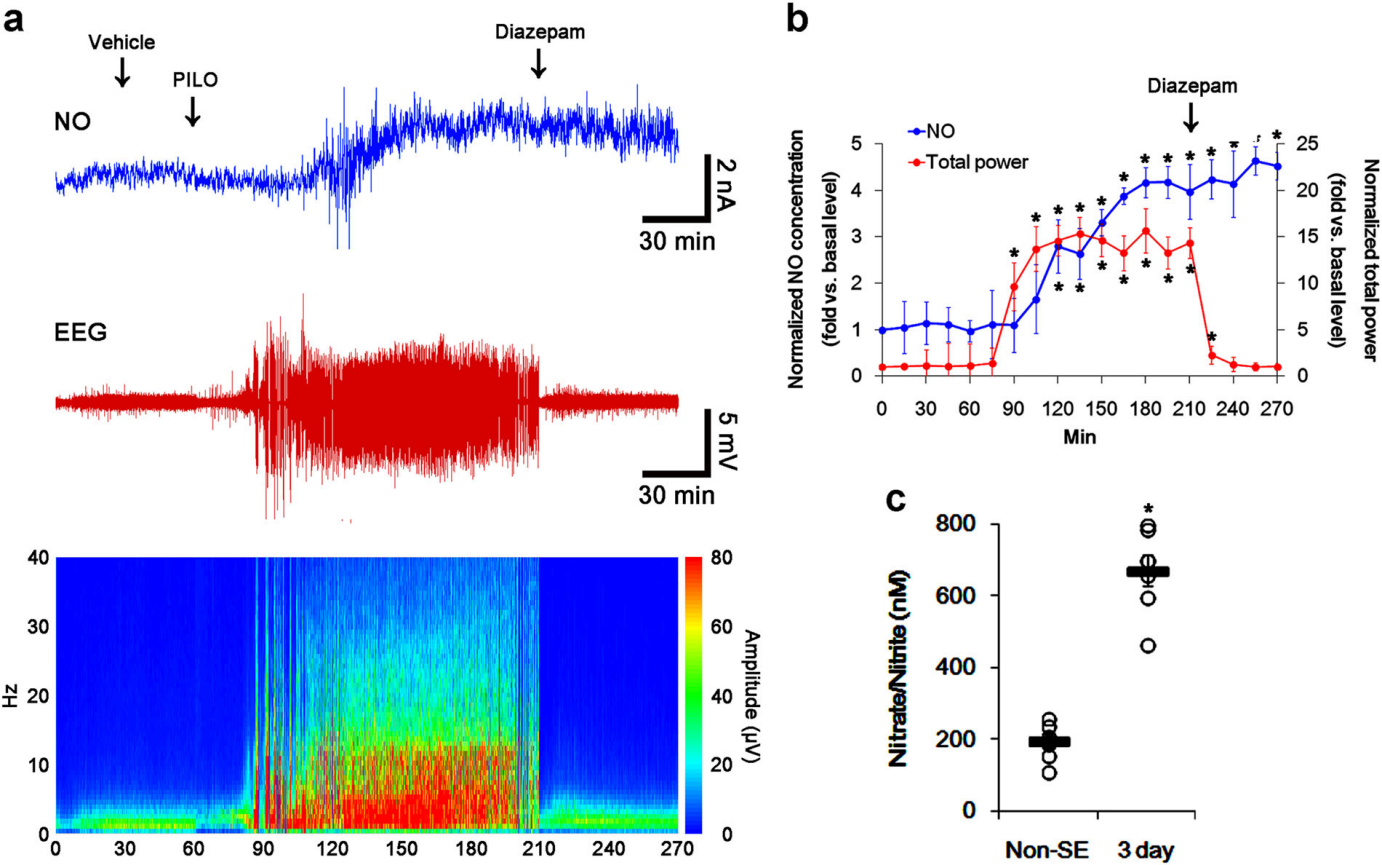
The role of seizure activity in NO generation in response to pilocarpine. Real-time simultaneous monitoring shows that NO level elevates after pilocarpine injection, and gradually increases during SE. Diazepam treatment recovers total EEG power to basal level, not NO level. Three days after SE, nitrate/nitrite concentration (NO products) is elevated, as compared to non-SE (control) animals. **a** Representative NO concentration (top), EEG trace (middle), and frequency-power spectral temporal maps (bottom). **b** Quantification of NO level and total EEG power in response to PILO (mean ± S.E.M.; *p < 0.05 vs. basal level; *n* = 7, respectively). **c** Quantitative values (mean ± S.E.M) of nitrate/nitrite levels in the hippocampus 3 days after SE. Open circles indicate each individual value. Horizontal bars indicate mean value. Error bars indicate SEM (*p < 0.05 vs. non-SE animals; *n* = 7, respectively)

**Fig. 3:**
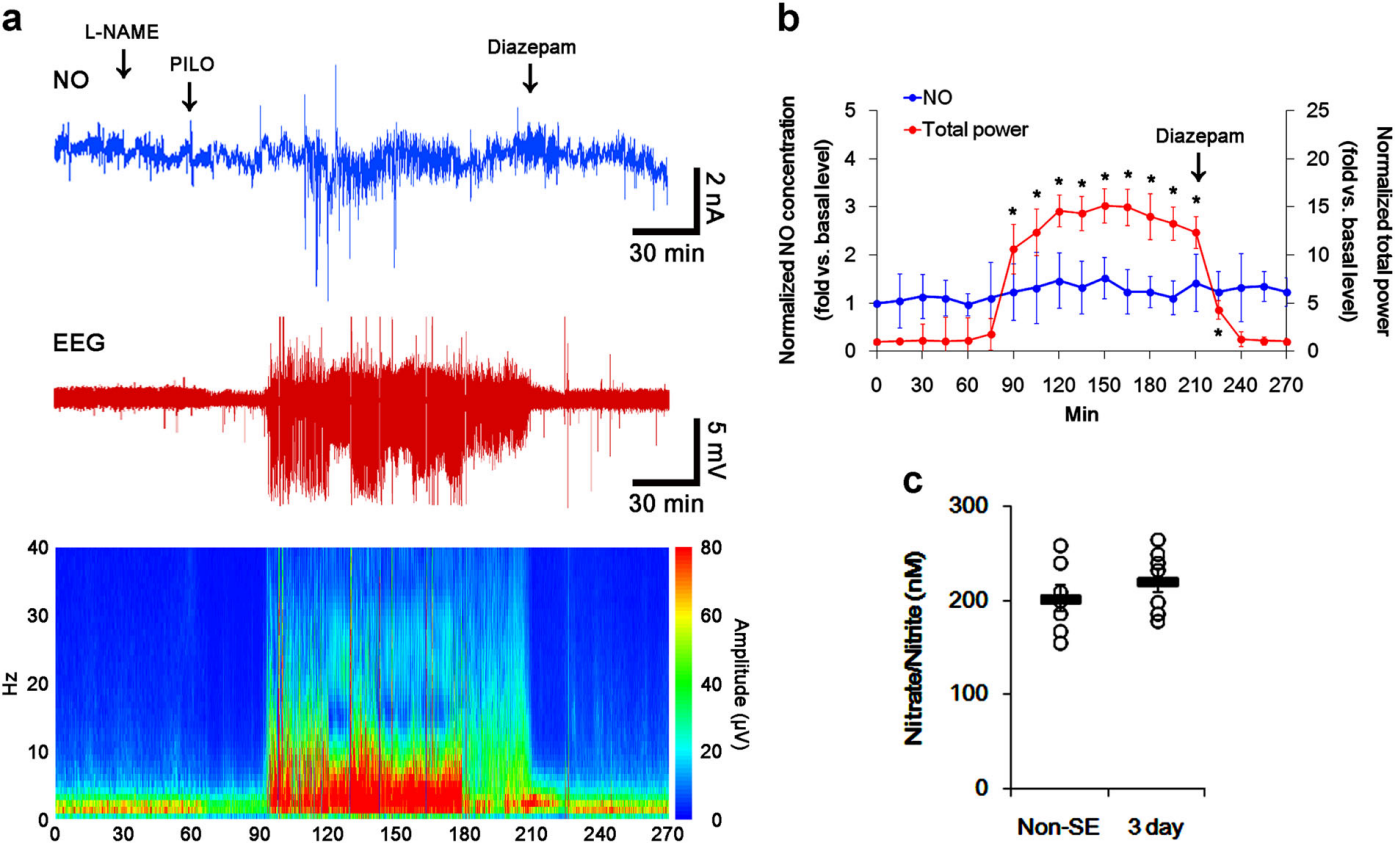
The effect of l-NAME on seizure activity and NO generation in response to pilocarpine. l-NAME effectively prevents the prolonged NO synthesis without altered seizure activity after pilocarpine injection. **a** Representative NO concentration (top), EEG trace (middle), and frequency-power spectral temporal maps (bottom). **b** Quantification of NO level and total EEG power in response to PILO (mean ± S.E.M.; *p < 0.05 vs. basal level; *n* = 7, respectively). **c** Quantitative values (mean ± S.E.M) of nitrate/nitrite levels in the hippocampus 3 days after SE. Open circles indicate each individual value. Horizontal bars indicate mean value. Error bars indicate SEM (*p < 0.05 vs. non-SE animals; *n* = 7, respectively)

In previous studies, we have reported that SE leads to activate signaling cascades for the NO synthesis, and increases NO metabolites^14,44^. To explore the relevance between NO synthesis and *S*-nitrosylation of DRP1 in CA1 neurons, we investigated the effect of l-NAME on SE-induced NO synthesis. In the present study, the real-time simultaneous monitoring of NO and EEG revealed seizure on-set and increase in NO level ~30 min and ~60 min after pilocarpine injection in vehicle-treated animals, respectively (*p* < 0.05 vs. basal level, respectively; Fig. 2a, b). NO concentration gradually increased during SE (*p* < 0.05 vs. basal level, Fig. 2a, b). Diazepam treatment effectively attenuated EEG total power to basal level, while it did not affect NO level (*p* < 0.05 vs. basal level, Fig. 2a, b). Consistent with our previous studies^14^, nitrate/nitrite concentration in the CA1 region was elevated to 3.4-fold of control (basal) level 3 days after SE (*p* < 0.05 vs. control animals, Fig. 2c). l-NAME prevented the prolonged NO synthesis, while it did not affect seizure activity in response to pilocarpine (Fig. 3a, b). In addition, nitrate/nitrite concentration was similar to that observed in control animals 3 days after SE (Fig. 3c). These findings indicate that NO may not have pro-convulsive or anti-convulsive effect, and that NO synthesis may be initiated by seizure on-set, but it may not be turn it off by seizure termination. Therefore, our findings suggest that prolonged NO synthesis may be involved in the diverse post-SE events independent of seizure activity.

### Reduced PDI expression impairs *S*-nitrosylation of DRP1 in CA1 neurons following SE, independent of prolonged NO production and thioredoxin 1 activity

**Fig. 4:**
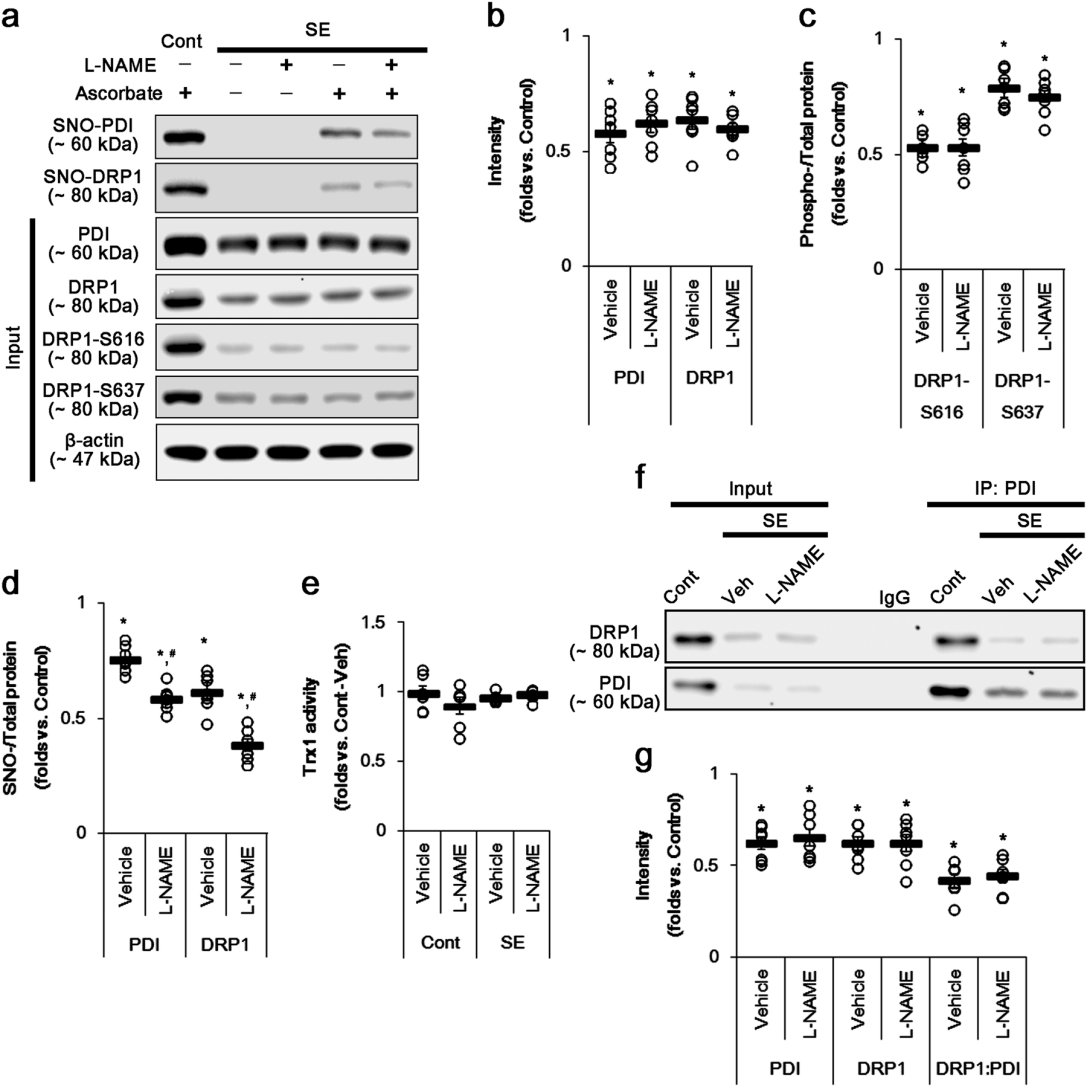
The effects of l-NAME on SNO-PDI, SNO-DRP1, Trx1 activity, and mitochondrial fusion in CA1 neurons 3 days after SE. SE significantly reduces expression levels of DRP1 and PDI, PDI-binding to DRP1, and phosphorylation levels of DRP1-S616 and S637 levels independent of Trx1 activity. Both SNO-PDI and SNO-DRP1 are more diminished by l-NAME treatment. **a** Representative western blot for the effect of l-NAME on expression, *S*-nitrosylation, and phosphorylation levels of PDI and DRP1 following SE. **b**–**d** Quantification of values (mean ± S.E.M) of expression, phosphorylation, and *S*-nitrosylation levels of PDI and DRP1. Open circles indicate each individual value. Horizontal bars indicate mean value. Error bars indicate SEM (*,^#^*p* < 0.05 vs. non-SE animals and vehicle, respectively; *n* = 7, respectively). **e** Quantification of values (mean ± S.E.M) of Trx1 activity. Open circles indicate each individual value. Horizontal bars indicate mean value. Error bars indicate SEM (*n* = 7, respectively). **f** Representative western blot for the effect of l-NAME on co-immunoprecipitation of PDI interaction with DRP1 following SE. **g** Quantification of co-immunoprecipitation analyses of PDI interactions with DRP1. Open circles indicate each individual value. Horizontal bars indicate mean value. Error bars indicate SEM (*p < 0.05 vs. non-SE animals; *n* = 7, respectively)

**Fig. 5:**
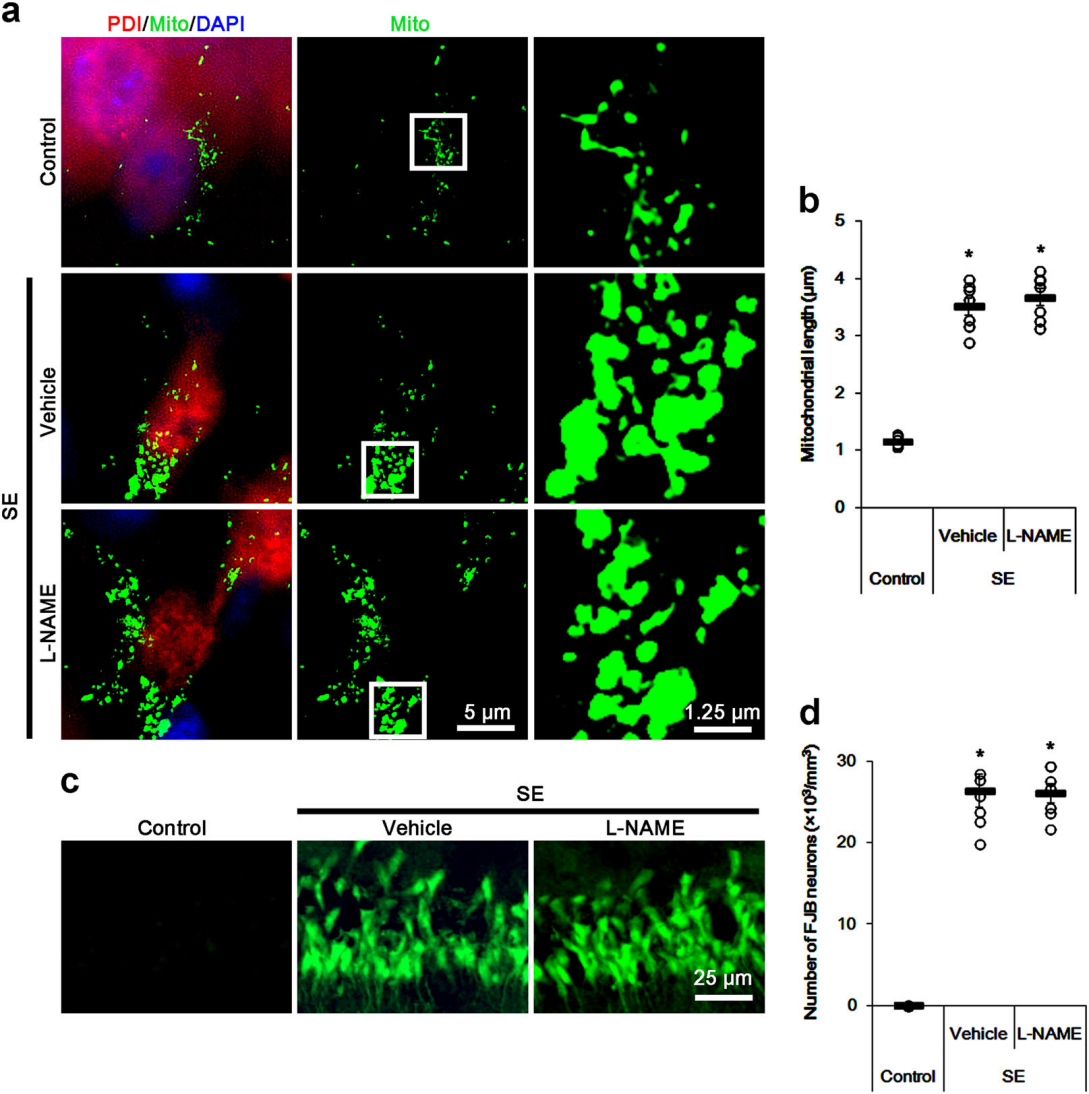
The effects of l-NAME on mitochondrial length and neuronal damage in CA1 neurons 3 days after SE. l-NAME do not affect SE-induced mitochondrial elongation and neuronal death in CA1 neurons. **a** Representative immunofluorescent photo for PDI and mitochondria (Mito) in CA1 neurons. The right panels are the high magnification photos of the rectangles in the middle panel. **b** Quantification of values (mean ± S.E.M) of mitochondrial length in CA1 neurons. Open circles indicate each individual value. Horizontal bars indicate mean value. Error bars indicate SEM (*p < 0.05 vs. vehicle; *n* = 7, respectively). **c** Representative photos for FJB staining CA1 neurons. **d** Quantification of values (mean ± S.E.M) of the number of FJB-positive CA1 neurons. Open circles indicate each individual value. Horizontal bars indicate mean value. Error bars indicate SEM (*p < 0.05 vs. vehicle; *n* = 7, respectively)

In the present study, l-NAME inhibited the mitochondrial fission via reducing DRP1-S616 phosphorylation and *S*-nitrosylation of DRP1 under normal condition (Fig. 1). Furthermore, SE led to the prolonged NO synthesis, which was abrogated by l-NAME (Figs. 2 and 3). Considering these data, it is likely that elevated NO concentration induced by SE would increase and *S*-nitrosylation of DRP1 and facilitate mitochondrial fission in CA1 neurons. Therefore, we explored how SE influences on *S*-nitrosylation of DRP1 in SE-induced CA1 neuronal death. In the present study, SE significantly reduced DRP1 expression in the CA1 region (*p* < 0.05 vs. control animals, Fig. 4a–d and Supplementary figure S2). Furthermore, SE decreased DRP1-S616 and DRP1-S637 levels in the CA1 region (*p* < 0.05 vs. control animals, Fig. 4a–d and Supplementary figure S2). However, DRP1-S616 level was diminished more than DRP1-S637 level following SE (*p* < 0.05 vs. control animals, Fig. 4a–d and Supplementary figure S2). Unexpectedly, SE decreased SNO-DRP1 level in the CA1 region (*p* < 0.05 vs. control animals, Fig. 4a–d and Supplementary figure S2), which was more reduced by l-NAME (*p* < 0.05 vs. vehicle, Fig. 4a–d and Supplementary figure S2). However, l-NAME did not affect DRP1 expression, DRP1-S616 and DRP1-S637 levels in the CA1 region following SE (Fig. 4a–d and Supplementary figure S2). On the other hand, thioredoxin 1 (Trx1) is one of the regulatory enzymes for *S*-nitrosylation. Trx1 denitrosylates the SNO-proteins by the conversion of its free C-thiol in active-site C32 and C35 to dithiol moiety^45^. Thus, we investigated whether impaired Trx1 activity is involved in these post-SE events. However, there was no difference in Trx1 activity among each group (Fig. 4e). Immunofluorescent study revealed that SE led to mitochondrial elongation and induced CA1 neuronal death, which was unaffected by l-NAME (Fig. 5a–d). These findings indicate that prolonged NO synthesis may not directly influence *S*-nitrosylation of DRP1 and mitochondrial dynamics in CA1 neurons following SE, although they were affected by l-NAME under physiological condition.

Consistent with our previous study^46^, we also found that SE significantly decreased PDI expression in CA1 neurons. Furthermore, SE abolished SNO-PDI level and PDI-binding to DRP1 in these neurons (*p* < 0.05 vs. control animals, Fig. 4a–d, f, g and Supplementary figure S2). l-NAME diminished SNO-PDI level in the CA1 region (*p* < 0.05 vs. vehicle, Fig. 4a–d, f, g and Supplementary figure S2), while it could not change PDI expression and PDI-binding to DRP1 following SE (Fig. 4a–d, f, g and Supplementary figure S2). Immunofluorescent study demonstrated that SE markedly reduced PDI expression in CA1 neurons (Fig. 5a). Since SNO-PDI acts as a NO donor^42^, our findings indicate that SE-induced reduction in PDI level may impair NO transport to DRP1 and affect *S*-nitrosylation and phosphorylation of DRP1, although SE evokes the sustained NO production. Taken together, our findings suggest the possibility that SE-induced downregulation of PDI may lead to mitochondrial elongation via impaired *S*-nitrosylation of DRP1 in CA1 neurons independent of Trx1 activity, in spite of prolonged NO production.

### PDI knockdown-reduced SNO-DRP1 and DRP1-S616 phosphorylation under normal condition

**Fig. 6:**
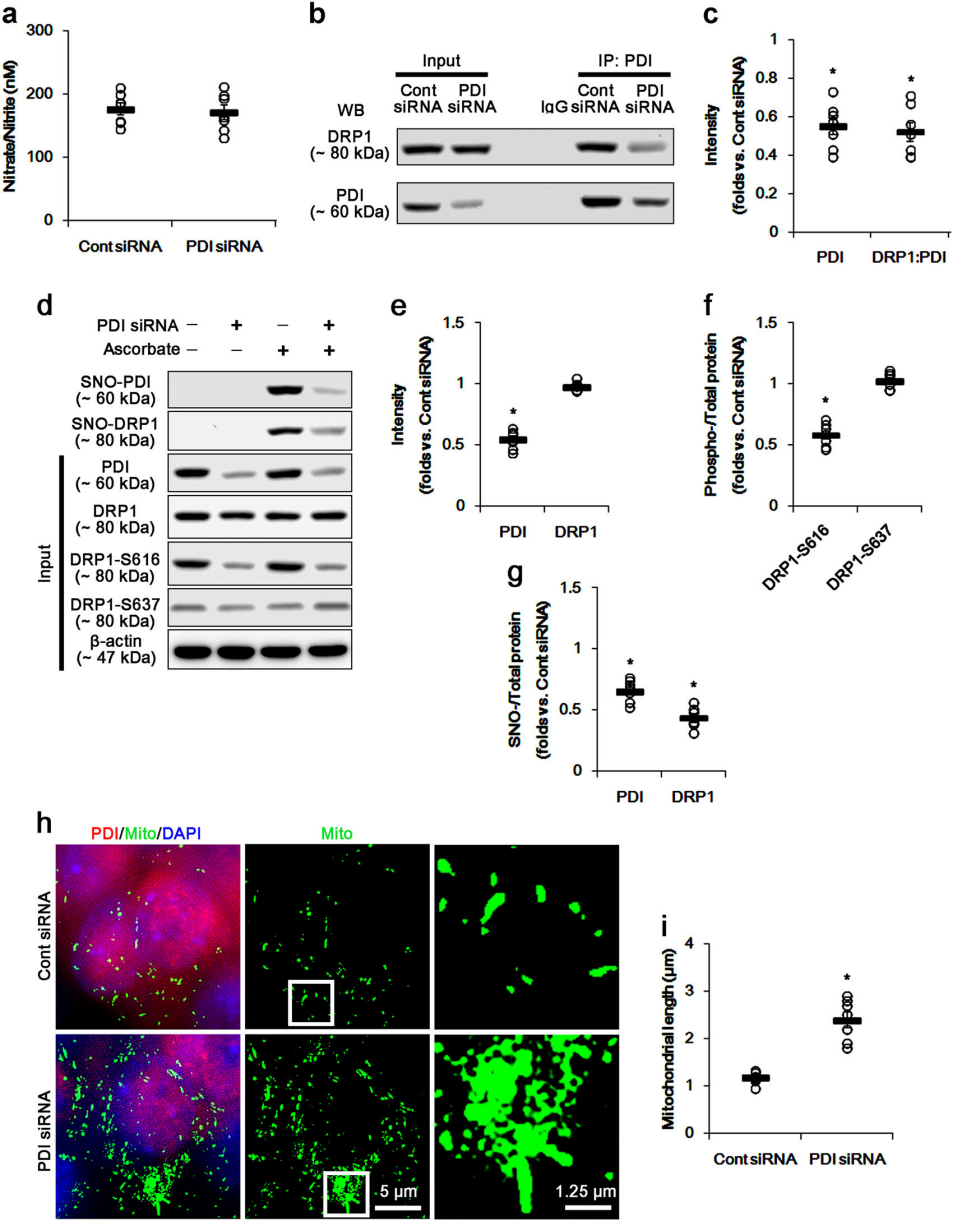
The effects of PDI knockdown on SNO-PDI, SNO-DRP1, and mitochondrial fusion in CA1 neurons under normal condition. PDI knockdown reduces PDI expression, the binding of PDI to DRP1, SNO-PDI, and SNO-DRP1 levels without changed DRP1 expression and nitrate/nitrite concentration. However, PDI knockdown elongates mitochondrial length in CA1 neurons, accompanied by elevated DRP1-S616 phosphorylation. **a** Quantitative values (mean ± S.E.M) of nitrate/nitrite levels in the hippocampus 7 days after PDI siRNA infusion. Open circles indicate each individual value. Horizontal bars indicate mean value. Error bars indicate SEM (*n* = 7, respectively). **b** Representative western blot for co-immunoprecipitation of PDI interaction with DRP1 following PDI knockdown. **c** Quantification of co-immunoprecipitation analyses of PDI interactions with DRP1 following PDI knockdown. Open circles indicate each individual value. Horizontal bars indicate mean value. Error bars indicate SEM (*p < 0.05 vs. control siRNA; *n* = 7, respectively). **d** Representative western blot for expression, *S*-nitrosylation, and phosphorylation levels of PDI and DRP1 following PDI knockdown. **e**–**g** Quantification of values (mean ± S.E.M) of expression, phosphorylation, and *S*-nitrosylation levels of PDI and DRP1 following PDI knockdown. Open circles indicate each individual value. Horizontal bars indicate mean value. Error bars indicate SEM (*p < 0.05 vs. control siRNA; *n* = 7, respectively). **h** Quantification of values (mean ± S.E.M) of mitochondrial length in CA1 neurons following PDI knockdown. Open circles indicate each individual value. Horizontal bars indicate mean value. Error bars indicate SEM (*p < 0.05 vs. control siRNA; *n* = 7, respectively). **i** Representative immunofluorescent photos for PDI and mitochondria (Mito) in CA1 neurons following PDI knockdown. The right panels are the high magnification photos of the rectangles in the middle panel

To confirm this hypothesis, we validated the effect of PDI knockdown on *S*-nitrosylation DRP1 under normal condition. PDI knockdown did not affect nitrate/nitrite concentration in the CA1 region (Fig. 6a). PDI knockdown-reduced PDI expression level and PDI binding to DRP1 level (*p* < 0.05 vs. control siRNA; Fig. 6b, c and Supplementary figure S3). Unexpectedly, PDI knockdown decreased SNO-DRP1, accompanied by reduced the amount of SNO-PDI. Furthermore, PDI knockdown abolished DRP1-S616 (not -S637) level without changed DRP1 expression level (*p* < 0.05 vs. control siRNA; Fig. 6d–g and Supplementary figure S3). Consistent with reduced DRP1-S616 phosphorylation, PDI knockdown resulted in mitochondrial elongation in CA1 neurons (*p* < 0.05 vs. control siRNA; Fig. 6h, i). These findings indicate that PDI may play a role as a NO transporter to DRP1, which may regulate *S*-nitrosylation-mediated DRP1-S616 phosphorylation. Thus, it is likely that SE-induced downregulation of PDI may evoke mitochondrial elongation via impaired *S*-nitrosylation of DRP1, in spite of prolonged NO production. Taken together our findings indicate that PDI may play a role as a NO transporter to DRP1, and that *S*-nitrosylation of DRP1 may accelerates the mitochondrial fission in the CA1 neurons by facilitating DRP1-S616 phosphorylation.

## Discussion

The major findings in the present study are that PDI may be one of NO donors for DRP1, and this PDI-mediated *S*-nitrosylation of DRP1 may facilitate mitochondrial fission via enhancing DRP1-S616 phosphorylation in CA1 neurons.

NO is a reactive free radical gas, plays a vital role in regulating many physiological and pathophysiological processes^45,47^. NO regulates many protein functions by means of *S*-nitrosylating proteins^48,49^. The *S*-nitrosylation of protein thiols is a redox-based post-translational modification that regulates protein functions and activities under both physiological and pathological conditions^50^. PDI is one of the initial ER chaperones^51,52^, which plays a crucial role in catalyzing disulfide bond formation, reduction, and isomerization^53–56^. However, *S*-nitrosylation of PDI inhibits its activity that modulates protein folding through thiol and disulfide bond formations. Thus, *S*-nitrosylation of PDI results in an increase in ubiquitinated and misfolded proteins, which contributes to neuronal cell death in various neurodegenerative disorders^38–41,57,58^. In a previous study^46^, we have reported that PDI expression is decreased in CA1 neurons 3 days after SE. Consistent with this report, the present data show that SE significantly reduced PDI expression in CA1 neurons, accompanied by diminished SNO-PDI level. The present study also revealed that l-NAME diminished SNO-PDI level in the CA1 region without changed PDI expression following SE. However, l-NAME could not attenuate SE-induced CA1 neuronal death. These findings indicate that downregulation of PDI expression, not *S*-nitrosylation of PDI, may play an important role in CA1 neuronal death induced by SE.

DRP1 is one of the key players for mitochondrial fission^6^. SE leads to mitochondrial elongation with reducing DRP1 expression and its S616/S637 phosphorylation ratio during programmed necrotic CA1 neuronal death^13^. The present data also reveal that SE decreased DRP1-S616 level more than its S637 level. Thus, these findings indicate that the net outcome in term of mitochondrial dynamics is determined by the ratio of DRP1 post-translation modifications at each phosphorylation site following SE. Interestingly, the *S*-nitrosylation of DRP1 also regulates its activity^25^. Indeed, DRP1 is rapidly phosphorylated at S616 and recruited to mitochondria upon exposure to the NO donor, *S*-nitrocysteine^30^. In the present study, we found that SE-reduced SNO-DRP1 and DRP1-S616 levels concomitant with mitochondrial elongation. Furthermore, l-NAME elongated mitochondrial length and decreased the levels of SNO-DRP1 as well as DRP1-S616 phosphorylation under physiological conditions. These findings indicate that *S*-nitrosylation of DRP1 may modulate DRP1-S616 phosphorylation, which facilitate mitochondrial fission.

On the other hand, the present study demonstrates that SE led to the prolonged NO synthesis. However, SE-reduced SNO-DRP1 and DRP1-S616 level without altering Trx1 activity. SE also decreased PDI expression and its binding to DRP1 in CA1 neurons. Furthermore, l-NAME diminished SNO-PDI level in these neurons without changing Trx1 activity under normal and post-SE conditions. These findings provide the possible PDI-mediated mechanism underlying *S*-nitrosylation and S616 phosphorylation of DRP1. This is because PDI acts as a denitrosylase, such as *S*-nitrosoglutathione reductase, Trx, and xanthine oxidase^59,60^. After denitrosylation of other proteins, PDI is *S*-nitrosylated by itself and acts as a NO transporter^42^. In the present study, reduction in PDI expression was induced by SE and PDI knockdown. Furthermore, PDI siRNA resulted in the reduction of SNO-DRP1 level and the mitochondrial elongation in CA1 neurons under normal condition. These findings indicate that PDI may be a NO donor for DRP1 rather than a denitrosylase, and that the reduced PDI expression induced by SE may impair the NO transport to DRP1, which may decrease S616 phosphorylation. Therefore, our findings indicate that PDI may be one of essential modulators for *S*-nitrosylation of DRP1 independent of Trx1 activity.

Recently, we have reported that PDI is one of endogenous reducing agents for disulfide bonds of C residues on NMDAR subunits in the hippocampus, and plays an important role in the modulation of ictogenesis and seizure susceptibility^34^. Briefly, PDI increases thiols on NMDAR, and PDI siRNA increases disulfide bond formation on this receptor under physiological condition^34^. Since NMDAR activation drives Ca^2+^ influx, which in turn activates neuronal NOS (nNOS)^61,62^, it is presumable that PDI siRNA would reduce SNO-DRP1 and DRP1-S616 levels by alleviating NMDAR functionality. In the present study, however, both l-NAME and PDI siRNA reduced the binding of PDI to DRP1, accompanied by the decreased SNO-PDI level. Therefore, our findings indicate that SNO-PDI may have a higher binding affinity to DRP1 than naive PDI, independent of NMDAR activity.

In the present study, we could not explain how *S*-nitrosylation of DRP1 facilitated DRP1-S616 phosphorylation and why l-NAME did not affect DRP1-S616 phosphorylation following SE, in spite of reductions in SNO-PDI and SNO-DRP1 levels. *S*-nitrosylation induces DRP1 dimerization, which directly stimulates DRP1 activity^25^. However, Bossy et al.^30^ have reported that *S*-nitrosylation do not affect DRP1 activity and formation of DRP1 dimers. Instead, NO influences DRP1-S616 phosphorylation by various kinase activations^30^. Therefore, the precise mechanism underlying *S*-nitrosylation-induced DRP1 phosphorylation remains unclear. Interestingly, DRP1-C644 residue within the GTPase effector domain of DRP1 is the critical *S*-nitrosylation site^25,27^, and influences its activity^63–67^. With respect to these previous studies, it is likely that *S*-nitrosylation of C644 may partially exert the allosteric changes of DRP1 to expose S616 sites. Indeed, *S*-nitrosylation induces the allosteric modification of various enzymes, channels, and receptors^68–71^. Further studies are needed to elucidate the underlying mechanisms of *S*-nitrosylation-mediated DRP1-S616 phosphorylation.

In conclusion, we found that the reduced PDI expression, not its *S*-nitrosylation, was involved in SE-induced neuronal death. Furthermore, PDI-mediated *S*-nitrosylation of DRP1 facilitated DRP1-S616 phosphorylation in CA1 neurons. Thus, we suggest that PDI may play an important role in mitochondrial dynamics via modulating *S*-nitrosylation of DRP1, independent of Trx1 activity.

## Materials and methods

### Experimental animals and chemicals

Male Sprague-Dawley (SD) rats (7-week-old) were used in the present study. All experimental protocols described below were approved by the Institutional Animal Care and Use Committee of Hallym University (Chuncheon, Republic of Korea) and all efforts were made to minimize animal suffering. All reagents were obtained from Sigma-Aldrich (St. Louis, MO, USA), except as noted.

### Measurement of NO synthesis and seizure activity

As described previously^72^, animals were anesthetized (urethane, 1.5 g/kg i.p.) and placed in a stereotaxic frame. Recording electrode (Plastics One Inc.) and NO sensor (ISO-NOPF200-L10, World Precision Instruments) were implanted into the left and right dorsal hippocampus (3.8 mm posterior; 2.0 mm lateral; 2.6 mm depth from bregma), respectively. The reference electrode was placed in the posterior cranium over the cerebellum. After recording a stable baseline for at least 30 min, animals were given pilocarpine (380 mg/kg i.p.) 20 min after atropine methylbromide (5 mg/kg i.p.). Some animals were given l-NAME (30 mg/kg, i.p.) 30 min prior to PILO injection. Total EEG power and NO concentration were measured during the 270-min recording session from each animal with with a DAM 80 differential amplifier (0.1–3000 Hz bandpass; World Precision Instruments) and Free radical analyzer (TBR4100, World Precision Instruments). Two hour after seizure on-set, diazepam (Valium; Hoffman la Roche, Neuilly sur-Seine, France; 10 mg/kg, i.p.) was administered. The data were analyzed using LabChart Pro v7 software (AD Instruments, NSW, Australia).

### PDI knockdown, l-NAME treatment, and SE induction

Rats were anesthetized with Isoflurane (3% induction, 1.5–2% for surgery, and 1.5% maintenance in a 65:35 mixture of N_2_O:O_2_). A brain infusion kit 1 (Alzet, USA) was implanted into the right lateral ventricle (1 mm posterior; 1.5 mm lateral; 3.5 mm depth from bregma) and connected to an osmotic pump (1007D, Alzet, USA) containing (1) control siRNA, (2) PDI siRNA, (3) vehicle, and (4) l-NAME (15 μg/μl), respectively. A PDI siRNA sequence corresponding to coding region (5′→3′) is sense: CUGCAAAACUGAAGGCAGAUU, and antisense: UCUGCCUUCAGUUUUGCAGUU. A non-silencing RNA was used as the control siRNA. The osmotic pump was subcutaneously placed in the interscapular region. Three days after surgery, some animals were treated with pilocarpine (380 mg/kg i.p.) 20 min after atropine methylbromide (5 mg/kg i.p.). Control animals received an equal volume of normal saline instead of PILO after the pretreatment with atropine methylbromide. Diazepam (Valium; Hoffman la Roche, Neuilly sur-Seine, France; 10 mg/kg, i.p.) was administered 2 h after onset of SE and repeated, as needed. Three days after SE, animals were used for western blot, co-immunoprecipitation, measurements of SHO-thiol or immunohistochemistry (see below).

### Nitrate/nitrite assay

Control animals and 3 days post-SE animals were implanted with a microdialysis probe (CMA 12) into the hippocampus (3.8 mm posterior; 2.0 mm lateral; 2.6 mm depth from bregma) under urethane anesthesia (1.5 g/kg, i.p.). The microdialysis probe was perfused with Ringer’s solution. The perfusion rate was 1 μl/min and efflux from the microdialysis probe was collected 240 μl. To measure nitrate/nitrite concentrations in perfusates, we used nitrate/nitrite assay kit (Cayman chemical company, USA), according to the manufacturer’s instructions.

### Western blot

The CA1 region in the hippocampus was dissected out and homogenized in lysis buffer (50 mM Tris containing 50 mM 4-(2-hydroxyethyl)-1-piperazineethanesulfonic acid (pH 7.4), ethylene glycol tetraacetic acid (pH 8.0), 0.2% Tergitol type NP-40, 10 mM ethylenediaminetetraacetic acid (pH 8.0), 15 mM sodium pyrophosphate, 100 mM β-glycerophosphate, 50 mM NaF, 150 mM NaCl, 2 mM sodium orthovanadate, 1 mM phenylmethylsulfonyl fluoride, and 1 mM dithiothreitol). Total protein content was measured by BCA protein assay kit. Western blotting was performed according to standard procedures. The primary antibodies were mouse anti-PDI (1:1,000, Abcam, ab2792), rabbit anti-DRP1 (1:1000, Thermo, PA1-16987), rabbit anti-phospho (p) DRP1-S616 (1:500, Cell Signaling, #4494), and rabbit anti-pDRP1-S637 (1:500, Cell Signaling, #4867). The rabbit anti-β-actin primary antibody (1:6000, Sigma, A5316) was used as internal reference. The signals were scanned and analyzed by ImageQuant LAS4000 system (GE health). The values of each sample were normalized with the corresponding amount of β-actin. The ratio of phosphoprotein to total protein was described as phosphorylation levels.

### Co-immunoprecipitation

The tissues were lysed in radioimmune precipitation buffer (RIPA) with protease and phosphatase inhibitor cocktails (Roche Applied Sciences) and 1 mM sodium orthovanadate. After calibration of total protein concentrations, and equal amounts of proteins were precipitated with the PDI antibody and subsequent protein G sepharose at 4 °C overnight^34^. Beads were collected, eluted in sample buffer and boiled at 95 °C for 5 min. Next, western blotting was performed according to standard procedures.

### Measurement of SNO-thiols

Modified biotin switch assay was performed with the *S*-nitrosylation Western Blot Kit (ThermoFisher) according to the manufacturer’s protocol. Briefly, lysates were reacted with ascorbate in HENS buffer for specific labeling with iodoTMTzero reagents with MMT pretreatment. Protein labeling can be confirmed by Western blot using TMT antibody. Thereafter, TMT-labeled proteins were purified by Anti-TMT Resin, eluted by TMT elusion buffer, and identified by Western blot according to standard procedures. For technical controls, we omitted ascorbate for each sample. The ratio of SNO-protein to total protein was described as *S*-nitrosylation levels.

### Trx1 activity assay

Tissues were homogenized in TE buffer. Total protein content was measured by BCA protein assay kit. To measure Trx1 activity in lysates, we used thioredoxin activity fluorescent assay kit (Cayman chemical company, USA), according to the manufacturer’s instructions.

### Immunohistochemistry

Rats were anesthetized with urethane anesthesia (1.5 g/kg, i.p.) and perfused transcardially with 4 % paraformaldehyde in 0.1 M phosphate buffer (PB, pH 7.4). Brains were post-fixed in the same fixative overnight and then cryoprotected and sectioned at 30 μm with a cryostat. Free-floating coronal sections were incubated in PDI antibody in PBS containing 0.3% Triton X-100 overnight at room temperature. Tissue sections were incubated with mixture of rabbit anti-mitochondrial marker (ATP synthase, 1:500 Abcam, ab176569)/rabbit anti-PDI (1:500, Abcam, ab2792) antibodies in PBS containing 0.3% Triton X-100 overnight at room temperature. Thereafter, sections were visualized with Cy2- and Cy3-conjugated secondary antibody. Immunoreaction was observed and analyzed using an Axio Scope microscope (Carl Zeiss) or a confocal laser scanning microscope (LSM 710, Carl Zeiss Inc., Oberkocken, Germany). To establish the specificity of the immunostaining, a negative control test was carried out with preimmune serum instead of the primary antibody. No immunoreactivity was observed for the negative control in any structures. All experimental procedures in this study were performed under the same conditions and in parallel^73^.

### Fluoro-Jade B staining

To analyze the neuronal damage, we applied Fluoro-Jade B (FJB) staining. Sections were placed on slides, dried, and immersed in 80% ethanol containing 1% sodium hydroxide. Next, samples were immersed in 70% ethanol solution for 2 min and in purified water for 2 min. After immersion in 0.06% potassium permanganate solution for another 10 min, samples were rinsed with purified water for 2 min. Images were captured using an AxioImage M2 microscope.

### Cell count and measurement of mitochondrial length

The CA1 subfield areas were delineated with a ×2.5 objective lens. The optical fractionator was used to estimate the number of FJB-positive neurons. The sampling procedure is accomplished by focusing through the depth of the tissue (the optical dissector height, *h*; of 15 μm in all cases for this study). The number of neurons cell type (*C*) in the CA1 subregions is estimated as: *C* = *ΣQ*^*−*^ × *t*/*h* × 1/asf × 1/ssf, where *Q*^*−*^ is the number of cells actually counted in the dissectors, ssf is the fraction of the sections sampled or section sampling fraction (of 1/6 in this study), and asf is the areal sampling fraction calculated by the area of the counting frame of the dissector (of 50×50 μm^2^ in this study)/the area associated with each *x*, *y* movement, grid (*x*, *y* step, 250×250 μm^2^ in this study). Individual mitochondrion length in CA1 neurons (*n* = 20/section) was measured by using ZEN lite software (Blue Edition, Carl Zeiss Inc., Oberkocken, Germany) following 3D-reconstruction: Based on our previous study^19,73^, 25 serial images (z-stack, 1 μm) were obtained from each hippocampal section. Serial images were stacked, alighned, visualized, and converted to 3D images using ZEN lite program. Thereafter, individual mitochondrial length (long axis) was measured. Two different investigators who were blind to the classification of tissues performed cell counts and measurement of mitochondrial length.

### Statistical analysis

Quantitative data are expressed as mean ± standard error of the mean. Data are analyzed by Student *t*-test or ANOVA followed by Newman–Keuls post hoc test. A *p* < 0.05 is considered to be statistically different.

## Electronic supplementary material

Supplementary information
