# Supplementary material for: PDI-mediated S-nitrosylation of DRP1 facilitates DRP1-S616 phosphorylation and mitochondrial fission in CA1 neurons
Source: Cell Death Dis. 2018 Aug 29;9(9):869. doi: 10.1038/s41419-018-0910-5 (PMC6115394; doi:10.1038/s41419-018-0910-5)
Supplement: Supplementary file 1 — Supplementary information [file 41419_2018_910_MOESM1_ESM.pdf]

Supplementary information

PDI-mediated *S*-nitrosylation of DRP1 facilitates DRP1-S616 phosphorylation and mitochondrial fission in CA1 neurons

Duk-shin Lee[1], Ji-Eun Kim[1,*]

[1]Department of Anatomy and Neurobiology, Institute of Epilepsy Research, College of Medicine, Hallym University, Chuncheon 24252, South Korea.

Running title: PDI-mediated *S*-nitrosylation of DRP1 in CA1 neuron.

* Correspondence should be addressed to J-EK (e-mail: jieunkim@hallym.ac.kr)

Fig. 1b

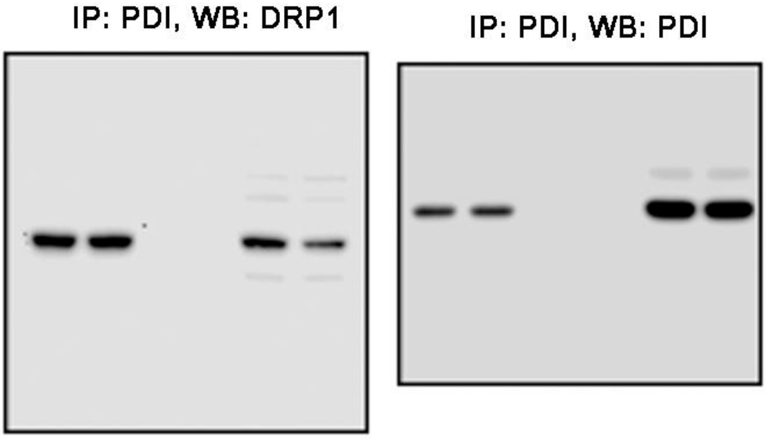

Fig. 1d

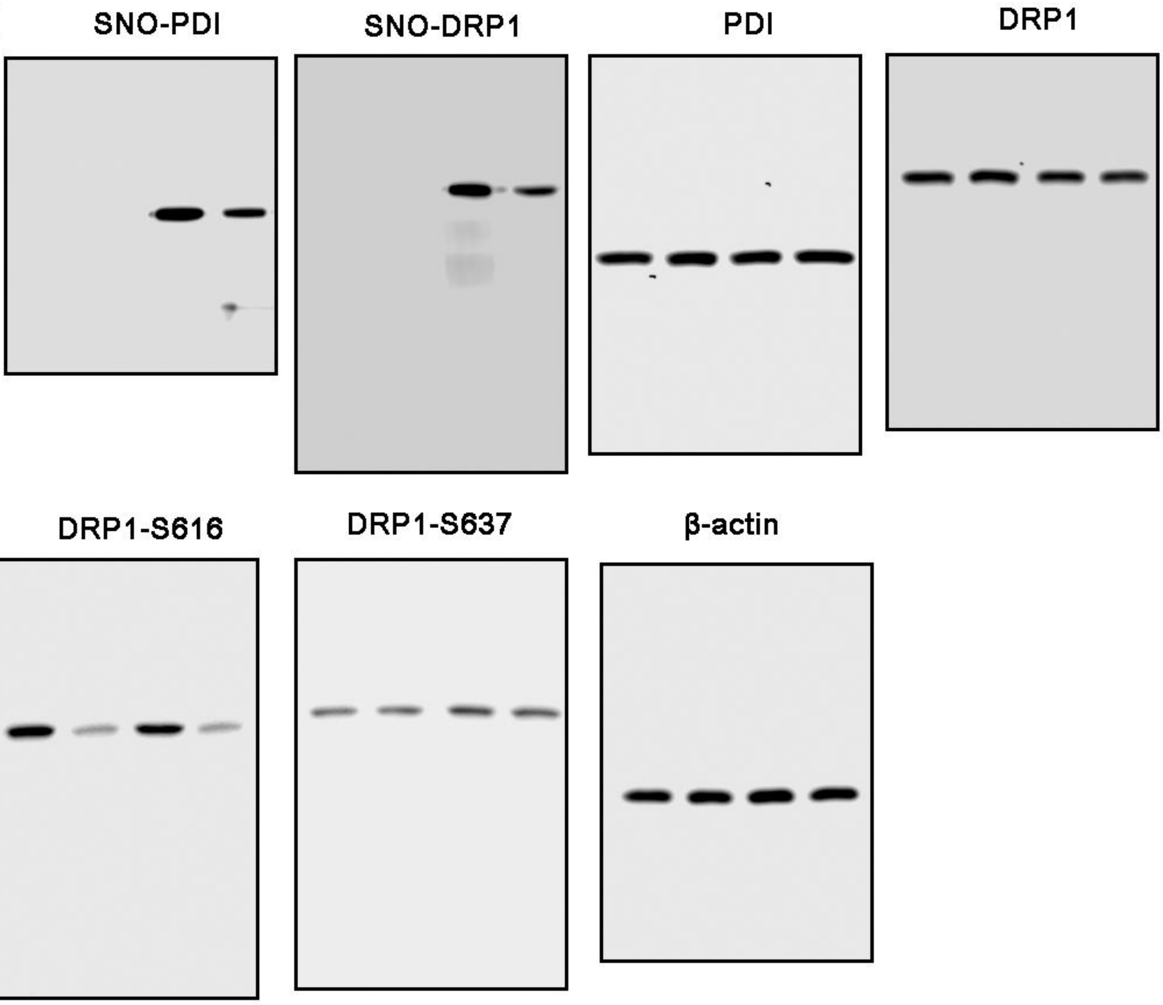

Supplementary Fig. 1. Full-length gel images of western blot data in Fig. 1.

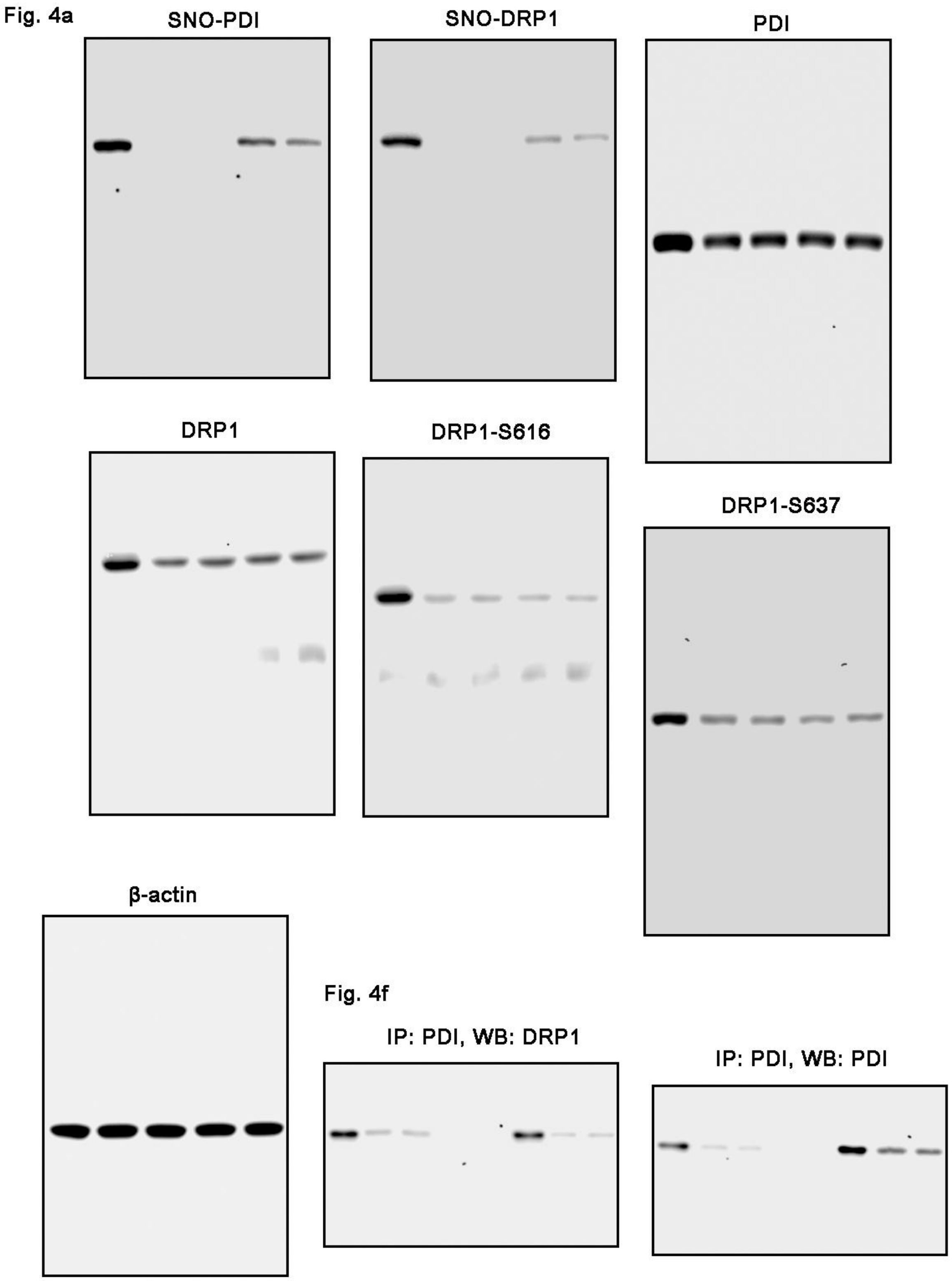

Supplementary Fig. 2. Full-length gel images of western blot data in Fig. 4.

Fig. 6b

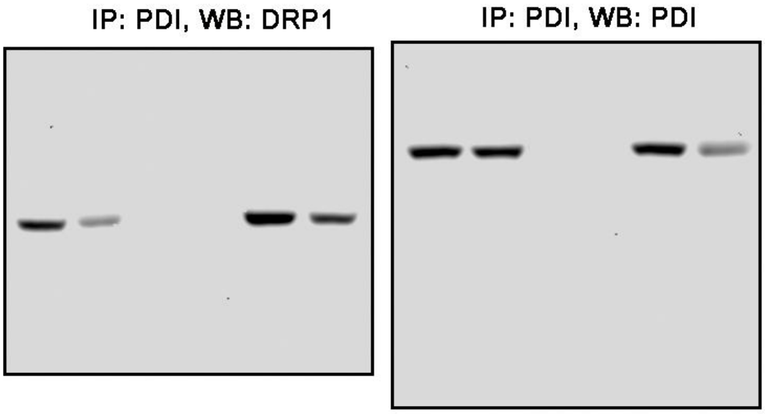

Fig. 6d

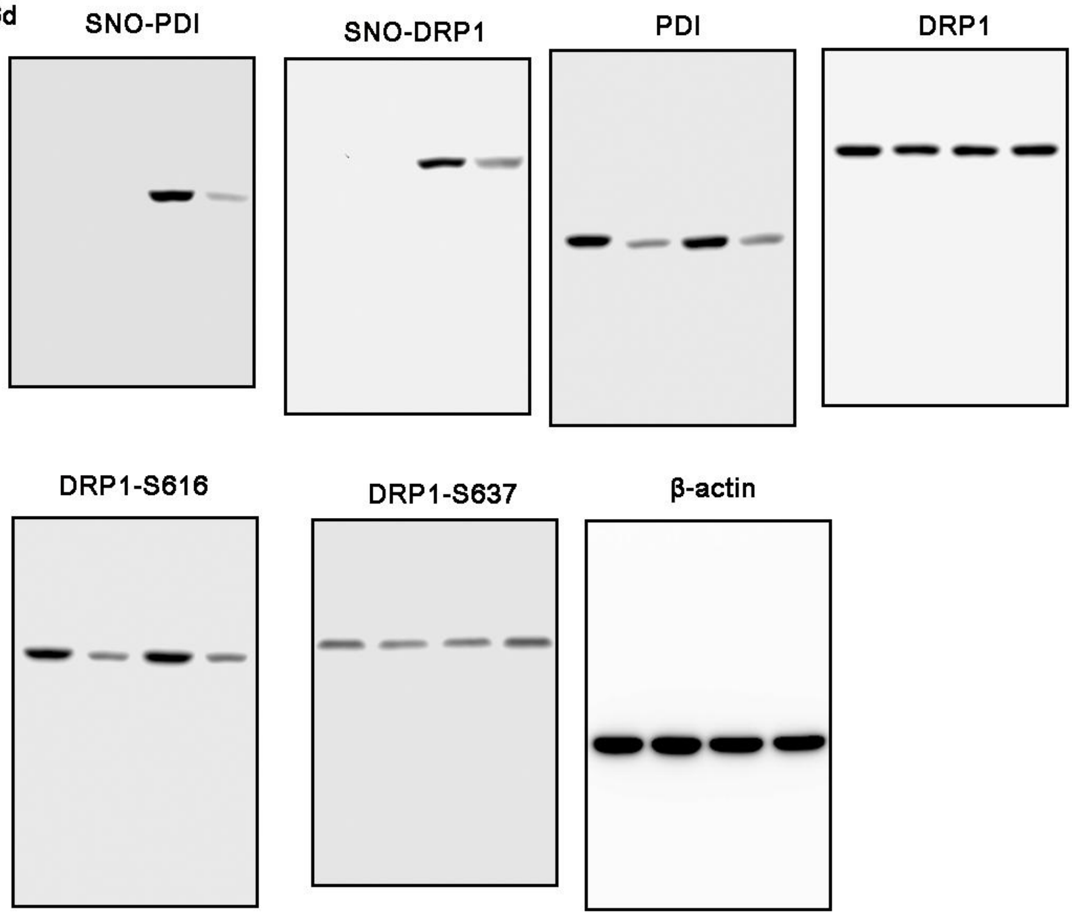

Supplementary Fig. 3. Full-length gel images of western blot data in Fig. 6.
